# Fine mapping of the major-effect QTL *qPHS4.1* revealed a *CsDOG1* gene controlling pre-harvest sprouting in cucumber (*Cucumis sativus* L.)

**DOI:** 10.3389/fpls.2025.1701268

**Published:** 2025-10-09

**Authors:** Mingming Cao, Qiang Deng, Huizhe Wang, Changyue Liu, Haiyan Zhao, Ruihuan Yang

**Affiliations:** State Key Laboratory of Vegetable Biobreeding, Cucumber Research Institute, Tianjin Academy of Agricultural Sciences, Tianjin, China

**Keywords:** cucumber, pre-harvest sprouting, *qPHS4.1*, CsDOG1, gene editing

## Abstract

Pre-harvest sprouting (PHS) in cucumber (*Cucumis sativus* L.) significantly reduces seed quality and yields in seed industry. The identification of PHS-associated quantitative trait loci (QTL) provides valuable genetic insights for improving PHS resistance in cucumber breeding practices. In this study, near-isogenic lines (NILs) targeting the major effect QTL locus were developed by the backcrossing of the highly PHS-sensitive donor parent P60 with the PHS-resistant recurrent parent Q12. PHS phenotypes of *qPHS4.1* ^Q12^ and *qPHS4.1* ^P60^ were consistent with the respective Q12 and P60, confirming that the *qPHS4.1* locus has a major-effect on PHS in cucumber. Using BC_4_F_2_ and BC_4_F_3_ populations, the *qPHS4.1* was fine-mapped to a 69.34 kb region, explaining 38.8% phenotypic variation. Through map-based cloning, *CsaV3_4G032930* (designated *CsDOG1*) was identified as the candidate gene, which encodes a DOG1 (Delay of Germination 1) domain-containing protein. Sequence analysis revealed that the mutation in P60 was a 3-bp deletion in the second exon of this gene (designated *Csdog1*), leading to a single amino acid deletion. Expression profiling revealed that *CsDOG1* exhibits strict seed-specific expression, with minimal transcripts in vegetative tissues. Promoter analysis demonstrated identical promoter sequence between Q12 and P60. The *cis*-acting regulatory elements, including several tissue-specific expression motifs, ABREs for ABA responsiveness and transcriptional promoter or enhancer elements were identified. Subcellular localization analysis revealed that the CsDOG1 protein was mainly localized in nucleus. Knockout of *CsDOG1* gene in the Q12 background resulted in mutants exhibiting extreme PHS susceptibility, confirming that *CsDOG1* is the causal gene responsible for PHS resistance underlying *qPHS4.1* locus. This study establishes a crucial theoretical foundation for elucidating the genetic mechanisms controlling PHS and offers valuable genetic resources for cucumber PHS-resistance breeding.

## Introduction

Cucumber (*Cucumis sativus* L.) is an economically important vegetable crop cultivated worldwide. In 2023, the cultivation area of cucumber was approximately 2,194,152 hectares worldwide (www.fao.org/faostat/en). As a primary agricultural commodity, the production of high-quality cucumber seeds is a critical prerequisite for seed dispersal and cultivation. PHS, also known as vivipary, is described as the premature germination of seeds within the maternal fruit and severely decreases the seed quality, yields and commercial viability of cucumber (Cao et al., 2021; Qu et al., 2023). It is well known that PHS corelated with seed dormancy in a complex and dynamic relationship, wherein high levels of dormancy maintain seed quiescence until the conditions are suitable for germination (Nishimura et al., 2018; Xu et al., 2019). On the other hand, a reduced or low level of dormancy can result in unwished early germination in the maternal plant (PHS sensitive) (Née et al., 2017). The investigation of genes underlying PHS is urgent for breeding PHS-resistant cucumber cultivars and advancing the understanding of molecular mechanisms controlling PHS. However, to date, there are few studies focusing on the genetic mechanisms responsible for PHS and seed dormancy in cucumber.

At present, extensive research has been performed on PHS and seed dormancy in many plants, such as cereal crops (Tai et al., 2021), *Arabidopsis* (Bentsink et al., 2006; Li et al., 2022), and tomato (Yao et al., 2020). Both PHS and seed dormancy are extremely complicated traits which are simultaneously regulated by numerous environmental, physiological and genetic factors. Based on genetic mapping, a list of quantitative trait loci (QTLs) associated with PHS and seed dormancy have been identified and many candidate genes underlying the QTLs were cloned. In wheat, ~200 QTLs associated with PHS and seed dormancy were identified on all of the 21 chromosomes (Kocheshkova et al., 2017; Zhou et al., 2017; Tai et al., 2021; Dhariwal et al., 2021; He et al., 2021; Gao et al., 2024). While more than 185 QTLs were detected in rice (Sohn et al., 2021). In barley, two significant QTLs, *Qsd1* and *Qsd2* were identified as major-effect QTLs controlling seed dormancy (Hickey et al., 2012; Nakamura et al., 2016; Hisano et al., 2022). The large number of QTLs suggests complicated genetic mechanisms responsible for PHS and dormancy in crops. Indeed, many well-known genes that control PHS and seed dormancy QTLs have been cloned, such as *TaMFT*, *TaPHS1*, *ThVp-1* and *TaMYB10* in wheat (Lang et al., 2021; Qu et al., 2023), *AHG1*, *SPL*, *OsSdt4*, *OsABI3*, *OsPHS8*, *OsVP1*genes in rice (Xu et al., 2019; Zhao B. et al., 2022; Qin et al., 2020; Chen et al., 2020), and *DOG1*, *AtABI3*, *AtABI5* genes in *Arabidopsis* (Bentsink et al., 2006; Dekkers et al., 2016). Abscisic acid (ABA) and gibberellins (GAs) are two most important phytohormones in determining seed dormancy and germination. Most of the genes have been proved to participate in the biosynthesis, catabolism and signaling pathways of ABA and GAs (Shu et al., 2016; Tuan et al., 2018; Zhao H. et al., 2022). The *DOG1* (*Delay of Germination 1*) gene was firstly identified in *Arabidopsis* using NILs (Bentsink et al., 2006; Fedak et al., 2016). *DOG1* can interact with ABA and provide primary dormancy for plant seeds (Zhao et al., 2015). Elevated *DOG1* expression promotes seed dormancy (Bentsink et al., 2006) while concurrently conferring PHS resistance in seeds. However, the genetic and molecular basis of seed dormancy and PHS in cucumber has not yet been fully elucidated.

PHS and seed dormancy in cucumber were controlled by multiple genes or QTLs (Cao et al., 2021; Qu et al., 2023). In our previous study, a major-effect QTL locus associated with PHS (*qPHS4.1*) was primarily located on cucumber chromosome 4 using the F_2_ population (Cao et al., 2021). However, relying exclusively on an F_2_ population for QTL mapping introduces methodological challenges and provides limited mapping resolution. Near-isogenic lines (NILs) serve as the ideal genetic resources for pinpointing candidate genes underlying target QTLs, as the confounding effects from genetic background variation on PHS phenotypic evaluation could be minimized (Wang et al., 2018; Xia et al., 2018). In this study, we developed backcrossing populations and generated a set of NILs with uniform genetic backgrounds differing only at the target *qPHS4.1* region. Using these genetic resources, we identified and functionally validated the causal gene *CsDOG1* in *qPHS4.1* locus. Our study provides novel insights into the molecular mechanisms controlling PHS and lays a foundation for cucumber PHS-resistance improving in seed production.

## Materials and methods

### Plant materials, population construction and phenotype evaluation

The two high-generation cucumber inbred lines, P60 and Q12 were used in the previous study (Cao et al., 2021). P60 is extremely sensitive to PHS and Q12 is highly PHS-resistant. Herein, to eliminate the complicated genetic background interference, *qPHS4.1* locus was introgressed from the donor parent P60, into the recurrent parent Q12. Firstly, P60 and Q12 were used as parent lines to generate the F_1_ (P60 × Q12). Then, Q12 was used as the recurrent parent and backcrossed with the F_1_ for four times, generating a series of backcrossing populations (BC_1_F_1_, BC_2_F_1_, BC_3_F_1_ and BC_4_F_1_ lines). For each backcrossing generation, two insertion and deletion (InDel) markers that flanked the *qPHS4.1* locus were employed for marker-assisted selection of heterozygous plants. Finally, the BC_4_F_1_ lines whose genomic background was almost similar to Q12 except for the substituted *qPHS4.1* allele were developed. The BC_4_F_2_ segregating population were generated by self-crossing of the BC_4_F_1_ lines. Homozygous lines, *qPHS4.1* ^P60^ and *qPHS4.1* ^Q12^ at the *qPHS4.1* region in agreement with P60 and Q12 were respectively selected as a NILs pair to validate *qPHS4.1* effect on PHS.

Plant growing conditions, farming management and PHS evaluation were conducted in accordance with our previous description (Cao et al., 2021). Briefly, two female flowers were reserved and pollinated. To assess PHS, fruits were harvested at 45 days after pollination (DAP), a stage when cucumber seeds were fully mature and any observed germination was a genuine PHS event. PHS rate (%) was calculated immediately after harvest as (germinated seeds/total seeds in one cucumber fruit) × 100%. The parent lines and the homozygous NILs were assayed with three biological replicates. The average PHS rates of two cucumber fruits grown on each maternal plant were calculated for further analysis.

### Marker development and fine mapping of *qPHS4.1*

Based on genome resequencing data, polymorphic InDel loci with multiple-base (≥10 bp) variations that could be amplified by PCR and separated on the 8% non-denatured polyacrylamide gel electrophoresis (PAGE) (Byun et al., 2009) were selected to develop codominant InDel markers. 13 codominant InDel markers located within the *qPHS4.1* interval were developed (detailed in **Supplementary Table S1** and **Supplementary Figure S1**) whose PCR products could be detected by 8% non-denatured PAGE and silver staining (Byun et al., 2009). The primer sequences were designed using Primer Premier 5.0 software. DNA was extracted from fresh leaves using DNAquick Plant System (Tiangen, Beijing, China). For convenience, the InDel-1, InDel-10 and InDel-15 markers were firstly employed to identify recombinant individuals from the BC_4_F_2_ plants at the seedling stage. Then, the identified recombinants were planted in the greenhouse and further genotyped by other markers. Although no suitable codominant InDel markers could be developed between InDel-4 and InDel-7, several significant InDel and SNP sites in this region were detected using the Hi-SNP high-throughput genotyping method (Shanghai Biowing Applied Biotechnology CO. LTD, Shanghai, China). Multiplex PCR and high-throughput sequencing genotyping were performed as previously described (Cao et al., 2021). Specific multiplex PCR primers of the markers were listed in **Supplementary Table S1**. QTL mapping of *qPHS4.1* was conducted using JoinMap 4.0 Van Ooijen (2006) with a maximum likelihood mapping algorithm and Kosambi mapping function, followed by QTL analysis in MapQTL 6 (Van Ooijen, 2009) via the Multiple-QTL Model (MQM) mapping algorithm at an LOD threshold > 3.0. The output logarithm of odds (LOD) scores along with the genetic map of the loci were graphed by MapChart 2.32 software (Voorrips, 2002). Based on QTL analysis, we selected 13 BC_4_F_2_ recombinant plants carrying crossovers between the flanking markers. These critical recombinants were advanced to generate independent BC_4_F_3_ families. For each BC_4_F_3_ family, individuals were subjected to marker identification and PHS phenotyping. We screened homozygous individuals from each BC_4_F_3_ family and classified them into two distinct categories, i.e., homozygous recombinants carrying crossover events within the interval and homozygous non-recombinants retaining intact parental haplotypes (either P60 or Q12). Each genotype category contained approximate 30 plants, which were grouped into three biological replicates for statistical analysis. Mean PHS rates for each type of homozygous plants in a BC_4_F_3_ family were calculated for fine mapping analysis.

### Candidate gene cloning and mutation analysis

Based on fine mapping results, genes located in the narrowed *qPHS4.1* region were annotated and functionally predicted according to Cucumber (Chinese Long) v3 Genome (http://cucurbitgenomics.org/v2/organism/19, Li et al., 2019) and Cucumber Multi-omics Database (http://www.cucumberdb.com) (Guan et al., 2024). Promoter sequences and the coding regions of the candidate gene were amplified from the two parents P60 and Q12 as well as 40 natural cucumber germplasms using specific primers. Sources and genotypes of the 40 cucumber germplasms are detailed in **Supplementary Table S3**. Sanger sequencing was performed by Beijing Tsingke Biotech Co., Ltd. Sequences alignment and mutation identification were performed by DNAMAN software. The cloned promotor sequences were analyzed on PlantCARE (Lescot et al., 2002) and New PLACE (Higo et al., 1999) websites.

### qRT-PCR analysis of *CsDOG1* gene located in *qPHS4.1*

At adult stage, true leaves, stems, roots, tendrils, unpollinated ovary and staminate flower of P60 and Q12 were sampled, respectively. After pollinated, cucumber cavity flesh and seeds of Q12 and P60 were separated and sampled at 25, 32 and 38 DAP, respectively. The samples were immediately frozen in liquid nitrogen for RNA extraction. Three biological replicates were taken for each sample. Total RNA was extracted using RNAprep Pure Plant Plus Kit following the manufacturer’s protocol (Tiangen, China). cDNA was synthesized using a RevertAid First Strand cDNA Synthesis Kit (Thermo Fisher Scientific Inc., MA, USA). The *tubulin* gene (GenBank ID: AF044573.1) was used as the reference gene to normalize relative gene expression values. qRT-PCR primers were detailed in **Supplementary Table S4**. The relative expression levels of *CsDOG1* were calculated using the 2^−ΔΔCt^ method (Livak and Schmittgen, 2001). Three technical replicates were conducted to each of the samples. Significant differences among the samples were checked by the Student’s *t*-test.

### Subcellular localization

To determine the subcellular location of CsDOG1 protein, full-length CDS of *CsDOG1* without the termination codon were ligated into the pBWA(V)HS vector to obtain a *35S*::*CsDOG1*::*GFP* fusion vector. A fused NLS::mKate protein was used as the nuclear marker (Zhao et al., 2017). Protoplasts were obtained from *Nicotiana benthamiana* leaves which were treated by enzymatic hydrolysis as described by Wang et al. (2024). Briefly, the sample was collected by centrifugation at 300 rpm for 3 min to remove the supernatant. The cells were washed twice using pre-cooling W5 solution. An appropriate amount of MMG solution was applied to resuspend the cells until the concentration reached 2×10^5^ cells·mL^-1^. 20 µL of DNA (10 µL of CsDOG1-GFP fusion vector and 10 µL of marker plasmid) were mixed into 200 μL of the protoplast suspension. Then, isovolumetric PEG4000 solution (220 μL) was gently added to resuspend the protoplasts and kept for 10-15 min at room temperature. The W5 solution was added to terminate the transformation, and the protoplasts were collected by centrifuging at 300 rpm for 3min. Finally, transformed protoplasts were resuspended in W5 solution and incubated at 25 °C for 10 h. Afterwards, the supernatant was removed. GFP and mKate fluorescence signals were examined using confocal microscopy (C2-ER, Nikon, Japan) with excitation at 488 nm and 561 nm, with emission at 510 and 580 nm, respectively.

### Gene editing plasmid construction and genetic transformation

To generate CRISPR/Cas9 gene editing vector, single-guide RNA (sgRNA) target sequences were designed based on the conserved site within the first exon of *CsDOG1*. Two independent 20 bp target sequences were selected for sgRNA design. The sgRNA expression cassettes were PCR-amplified and then purified by 1.5% agarose gel electrophoresis and TIANgel Purification Kit (Tiangen Biotech, Beijing). Subsequently, the sgRNA expression cassettes were assembled into a modified gene editing vector K6-KRSN-ccdb based on CRISPR/Cas9 system using the Golden Gate ligation method with BsaI/Eco31I restriction enzyme and T4 DNA ligase. The positive recombinant plasmid was transformed into *Agrobacterium tumefaciens* strain EHA105. *Agrobacterium*-mediated transformation of cucumber line Q12 was accomplished using the cotyledon transformation method by Wuhan Boyuan Biotechnology. Genomic DNA of T_0_ and T_1_ plants was PCR-amplified using specific primers (listed in **Supplementary Table S4**). Mutation sites were examined by Sanger sequencing. Homologous T_1_ mutants were selected for PHS phenotyping analysis.

## Results

### Mapping population construction and phenotypic evaluation

The PHS-sensitive inbred line P60 was used as maternal parent and crossed with the highly resistant inbred line Q12. The F_1_ hybrid (P60×Q12) was backcrossed with Q12 for four times to generate the backcrossing populations. In our previous study, we performed QTL-seq analysis using F_2_ populations derived from the same P60×Q12 cross. Through phenotypic data collected over two growing seasons, the major-effect *qPHS4.1* locus was mapped on chromosome 4 (Cao et al., 2021). The flanking markers InDel-1 and InDel-2, which delineated the entire *qPHS4.1* region, were utilized for marker-assisted selection during each backcross generation. The BC_4_F_1_ lines were generated after four times of backcrossing and then self-crossed to develop the BC_4_F_2_ segregating population that contains 3190 individuals. Five homozygous *qPHS4.1* ^P60^ lines and seven homozygous *qPHS4.1* ^Q12^ lines were selected from the BC_4_F_2_ plants as a set of NILs to validate the effect of *qPHS4.1* locus on PHS. Meanwhile, the identified recombinant BC_4_F_2_ individuals were selected to fine map the *qPHS4.1* locus.

**Figure 1:**
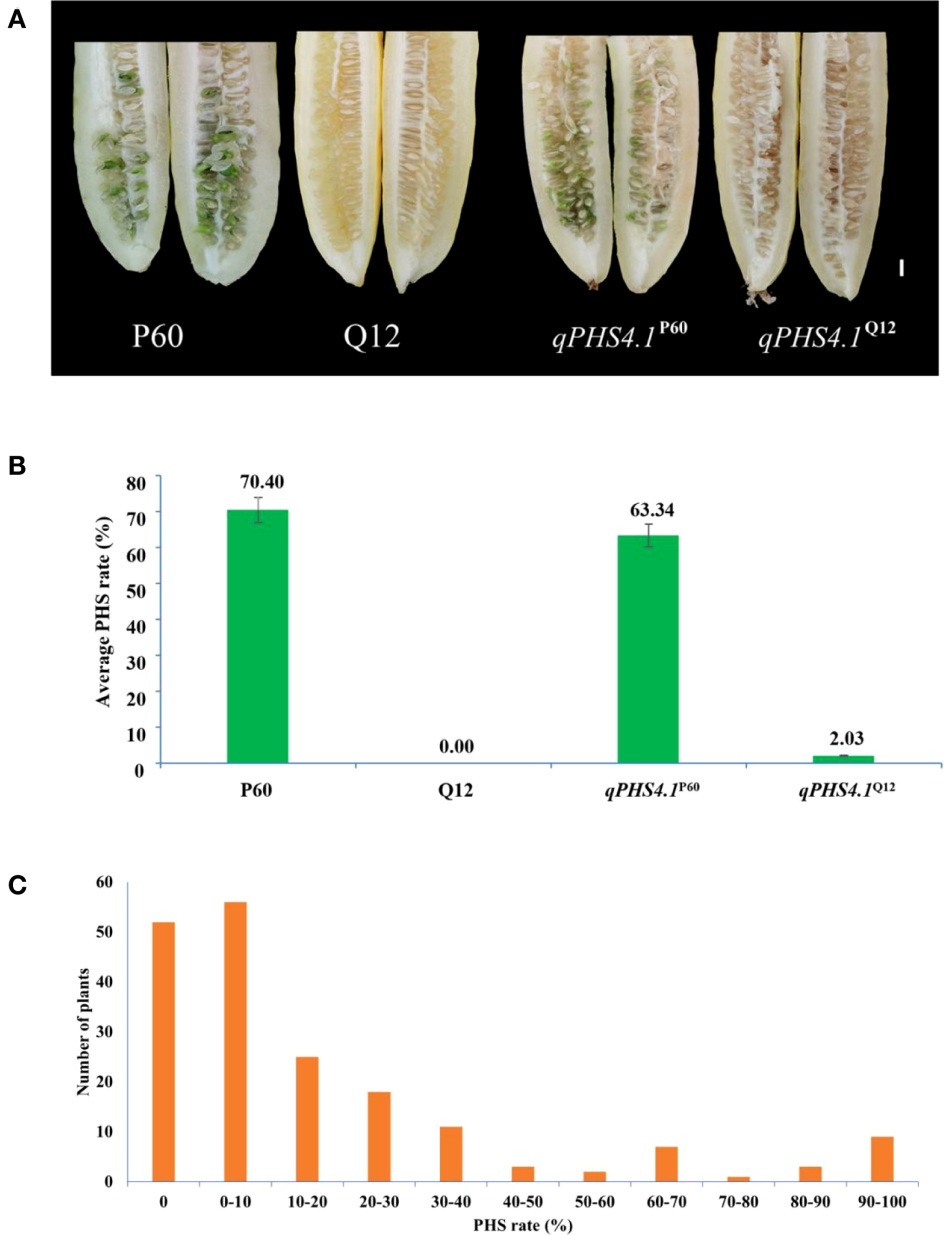
PHS phenotypic characteristics of the parental lines and NILs, and PHS frequency distribution in BC_4_F_2_ population. **(A)** PHS phenotypes of the parental lines and NILs, Scale bar = 1 cm; **(B)** PHS phenotypic data of the parental lines and NILs, Data were shown as means ± SD; **(C)** Frequency distribution of BC_4_F_2_ population.

Based on PHS phenotypic evaluation, mean PHS rate of the *qPHS4.1* ^P60^ lines was 63.34%, whereas the donor parent P60 was 70.40% and no significant difference was observed between *qPHS4.1* ^P60^ and P60 (*p* > 0.05, *t*-test, hereinafter). Q12 showed complete resistance to PHS. On the contrary, PHS rates of the NIL *qPHS4.1* ^Q12^ lines varied from 0% to 4.18% (only 2.03% on average), with no obvious differences from the recurrent parent Q12 (*p* > 0.05) (**Figures 1A, B**).

### Marker development and fine mapping of *qPHS4.1*

The almost parallel PHS phenotypes between the NILs and their parental lines revealed that *qPHS4.1* was the major-effect QTL locus controlling PHS trait in cucumber. To further refine the location and conduct map-based cloning of the candidate gene(s) in *qPHS4.1* locus, we developed 13 codominant InDel markers based on polymorphic loci with multiple base variations. Due to the absence of suitable codominant InDel markers between InDel-4 and InDel-7, we genotyped all the significant polymorphic sites in this interval via Hi-SNP high-throughput genotyping method.

Firstly, three markers (InDel-1, upstream side of *qPHS4.1*; InDel-10 in the middle; InDel-15 at downstream side) were subjected to identify segregating recombinants from the BC_4_F_2_ population at the seedling stage. The BC_4_F_2_ individuals exhibiting different genotype combinations at these three sites were identified as recombinants. Otherwise, individuals showing identical genotypes were classified as non-recombinant. Consequently, from the 3190 BC_4_F_2_ individuals, we identified 187 recombinant plants, which were advanced to greenhouse trials for high-resolution genotyping and detailed PHS characterization.

**Figure 2:**
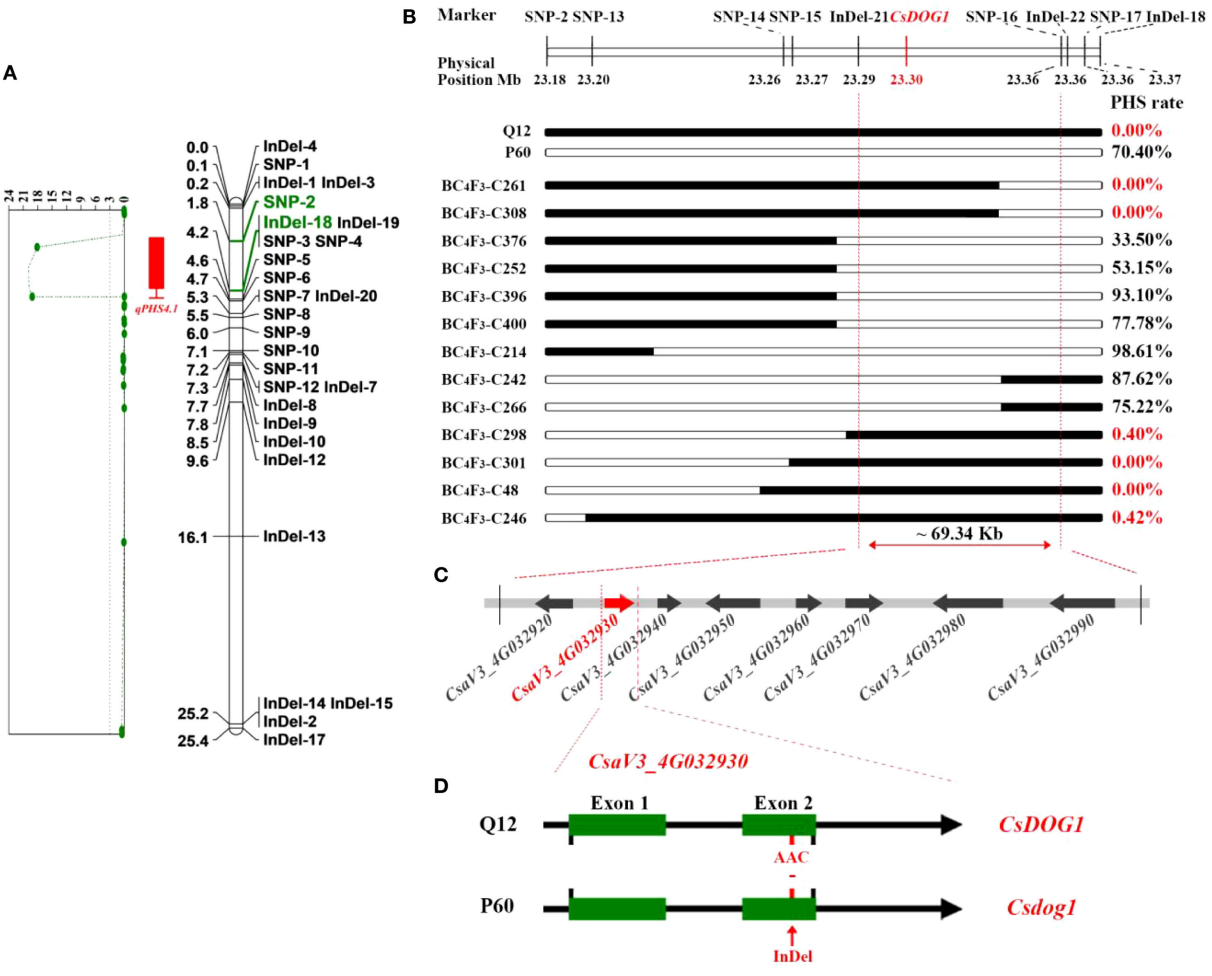
Fine mapping of the *qPHS4.1*. **(A)** QTL mapping indicates that *qPHS4.1* is located between SNP-2 and InDel-18 by the LOD values exceeded 3.0; **(B)** Fine mapping by homozygous recombinant BC_4_F_3_ families. Black and white segments indicate the homozygous genomic segments from Q12, P60, respectively. PHS rate exhibits the mean phenotypic data for each BC_4_F_3_ family; **(C)** Eight putative genes located in the ~ 69.34 kb genomic region according to the cucumber Chinese long v3 reference genome. **(D)** Gene structure and allelic mutation in the candidate *CsaV3_4G032930* gene between Q12 and P60. Green boxes represent the exons. InDel mutant site occurred in Exon 2.

Secondly, the markers in *qPHS4.1* region were subsequently employed to genotype the 187 recombinant BC_4_F_2_ individuals. Genotypes consistent with Q12 were recorded as “A”, while those consistent with P60 were recorded as “B”, and the hybrid genotypes were assigned as “H” (same as hereafter; detailed in **Supplementary Table S2**). Based on the genotypes generated by the markers, the regional genetic linkage map for *qPHS4.1* was constructed by JoinMap 4.0 software (Van Ooijen, 2006). The PHS rates among the 187 recombinant individuals exhibited a broad phenotypic spectrum (0-100%), with a mean incidence of 17.44%. (**Figure 1C**; **Supplementary Table S2**). The observed continuous frequency distribution of PHS rates among recombinants supports a quantitative genetic control for PHS in cucumber. Combining the PHS phenotype data with the genetic linkage map, we performed QTL analysis using MapQTL6. With a threshold of LOD values ≥ 3.0, the *qPHS4.1* locus was narrowed down to a ~ 184.39 kb interval franking by SNP-2 and InDel-18 markers (**Figure 2A**), explaining 38.8% of the phenotypic variation for cucumber PHS.

To further fine-map the *qPHS4.1* locus, we screened BC_4_F_2_ plants for recombination events within the interval defined by SNP-2 and InDel-18. This screening identified 13 critical recombinant individuals, which were subsequently self-pollinated to establish distinct BC_4_F_3_ families. To achieve higher mapping resolution, we developed seven additional markers within this interval. These new markers were employed to genotype the individuals within each BC_4_F_3_ family. Homozygous recombinants were identified from each BC_4_F_3_ family and subjected to PHS phenotyping. The PHS phenotype for homozygous recombinant plants in each BC_4_F_3_ family was determined by calculating the mean PHS rate (**Figure 2B**; **Supplementary Table S6**). By integrating genotyping data with a comparison of PHS phenotypes between homozygous recombinants and their corresponding non-recombinants within a BC_4_F_3_ family, we delineated the *qPHS4.1* locus to a ~69.34 kb critical region flanked by InDel-21 and SNP-16 (**Figure 2B**).

### Candidate gene identification and genetic alteration analysis

According to cucumber reference genome, only eight genes were annotated within the narrowed 69.34 kb region (**Figure 2C**). Comparative analysis of resequencing data from P60 and Q12 detected three mutation sites within this interval. Among them, one mutation was a 3-bp InDel in the coding region of *CsaV3_4G032930* gene, and the other two mutations occurred in the upstream of other genes. Based on gene annotation, *CsaV3_4G032930* gene was predicted to encode a DOG1 (Delay of Germination 1) domain-containing protein and considered as the candidate gene for controlling PHS in cucumber. The *DOG1* gene, originally identified in *Arabidopsis* as a master regulator of seed dormancy (Bentsink et al., 2006; Carrillo-Barral et al., 2020; Li et al., 2022), shares high sequence similarity with the cucumber gene *CsaV3_4G032930*. We annotated *CsaV3_4G032930* as *CsDOG1* in cucumber based on its homology to *Arabidopsis DOG1*. The PHS-sensitive parent P60 carries a 3-bp deletion in this gene, which we designated as *Csdog1*. Gene structure analysis revealed that *CsDOG1* gene (*CsaV3_4G032930*) comprises two exons, with the 3-bp deletion in *Csdog1* allele located in Exon 2 (**Figure 2D**; **Supplementary Figure S2A**). The 3-bp deletion in *Csdog1* is predicted to cause an in-frame deletion of a single asparagine residue (-N) in the encoded protein of P60 (**Supplementary Figure S2B**).

To validate the association between the 3-bp InDel mutation and PHS phenotype, we cloned and sequenced the full-length of *CsDOG1* genomic region from 40 diverse cucumber accessions along with parental lines Q12 and P60. Sequence analysis confirmed the presence of the 3-bp InDel polymorphism within the target genomic region. Subsequently, phenotypic and genotypic analysis revealed two distinct groups: (1) 11 accessions with “A” genotype showing minimal PHS rates (PHS rates ranged from 0% to 4.11%; mean: 0.91%), and (2) 25 lines with “B” genotype exhibiting significantly higher PHS rates (ranged: 5.72-90.91%; mean PHS rate: 45.47%) (**Table 1**; **Supplementary Table S3**). Additionally, in the 187 BC_4_F_2_ recombinants, the average PHS rate of “A” genotype plants was 0.23%, while that of “B” plants was 47.59%. The phenotypic and genotypic analyses of the natural cucumber accessions consistently corroborated the recombinant BC_4_F_2_ data (**Table 1**), providing strong evidence for the association between the 3-bp InDel mutation in *CsDOG1* and PHS variation.

**Table 1:** Summary of numbers and PHS rates for the polymorphic 3-bp InDel in recombinant BC_4_F_2_ plants and Natural Cucumber Lines.

Penotype	BC_4_F_2_				Natural cucumber lines		
	A	H	B	χ^2^ (1:2:1)	A	H	B
Number of plants	49	93	45	0.06	11	4	25
Average PHS rate ± SD (%)	0.23 ± 0.52	12.46 ± 14.66	47.59 ± 34.03		0.91 ± 1.20	16.86 ± 15.09	45.47 ± 27.40

### Expression pattern of *CsDOG1* and promoter analysis

**Figure 3:**
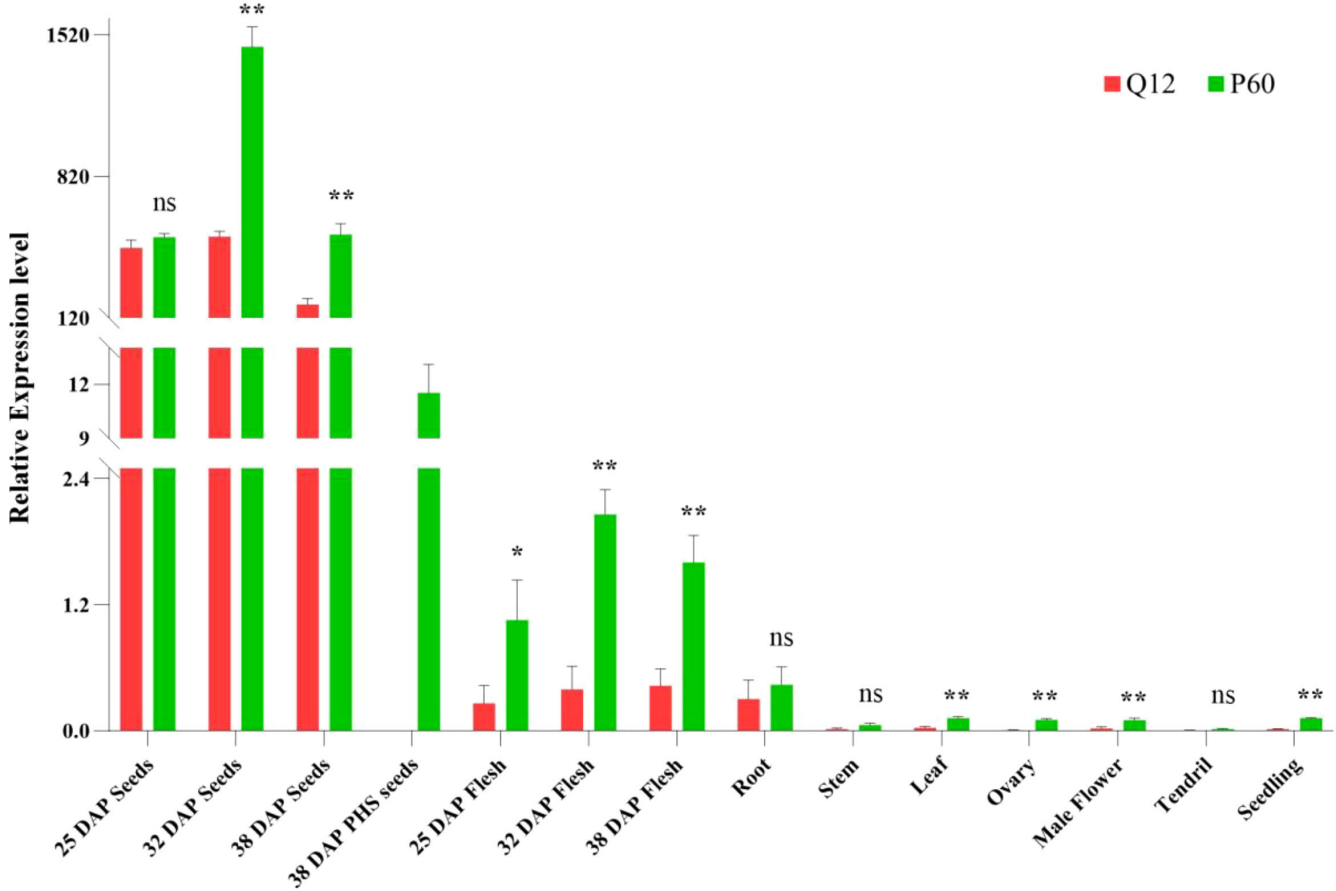
Relative expression profiles of *CsDOG1* gene in different tissues between Q12 and P60. *CsDOG1* gene is significantly higher expressed in the ungerminated seeds, but much lower or nearly no expression in the other tissues, including cucumber flesh. 38 DAP PHS seeds are the PHS seeds in P60. No seed germinated within Q12 fruit. Significance difference between Q12 and P60 was conducted by Student’s *t*-tests (ns: no significant difference; *p: < 0.05; **p: < 0.01).

We isolated the developing or matured seeds from Q12 and P60 fruits as along with their maternal flesh to analyze the expression levels of *CsDOG1* at 25, 32 and 38 DAP stages, respectively. Consistent with expectations, PHS was observed in P60 at 38 DAP, and the germinated P60 seeds were collected for analysis. We further characterized the expression patterns of *CsDOG1* across different tissues, including seedlings, roots, stems, leaves, pre-anthesis ovary and male flower, and tendril at the adult-plant stage. *CsDOG1* exhibits significantly higher expression in seeds compared to other tissues, where the transcript levels are either minimal or undetectable (**Figure 3**), demonstrating seed-specific expression. During seed developmental stages, *CsDOG1* transcript levels increased significantly from 25 DAP to 32 DAP, followed by a marked decline in subsequent stages. No significant difference in *CsDOG1* expression was observed between Q12 and P60 seeds at 25 DAP. However, significantly higher expression levels were detected in P60 compared to Q12 seeds from 32 to 38 DAP (*p* < 0.01). The germinated P60 seeds exhibited significantly lower *CsDOG1* expression compared to the ungerminated seeds, suggesting that germination could terminate the transcription of *CsDOG1* (**Figure 3**). These results demonstrated that *CsDOG1* is a seed-specific gene in cucumber, consistent with the known *Arabidopsis DOG1* function (Bentsink et al., 2006; Nakabayashi et al., 2012).

To investigate the molecular basis of *CsDOG1*’s seed-specific expression, we cloned and analyzed a 2,048 bp promoter region upstream of the translation start site (ATG) from both parental lines (Q12 and P60) for putative *cis*-regulatory elements. Sequence alignment revealed 100% conservation of the promoter region between Q12 and P60 (**Supplementary Figure S3**). Bioinformatic analysis identified multiple conserved *cis*-acting elements in the *CsDOG1* promoter, including core promoter elements (TATA-box and CAAT-box) and other putative transcription factor binding sites known to enhance basal transcription. Furthermore, we identified multiple functional cis-elements in the CsDOG1 promoter region (**Supplementary Table S5**), including: (i) tissue-specific motifs: GATA-box (light responsiveness), Canbnnapa (endosperm expression), and AACAcoreosglub1 (seed-specific regulation); (ii) ABA-responsive elements (ABREs): Two ACGTGGC motifs located at -140 bp and -156 bp upstream of the ATG start codon.

### Subcellular localization of CsDOG1 protein

**Figure 4:**
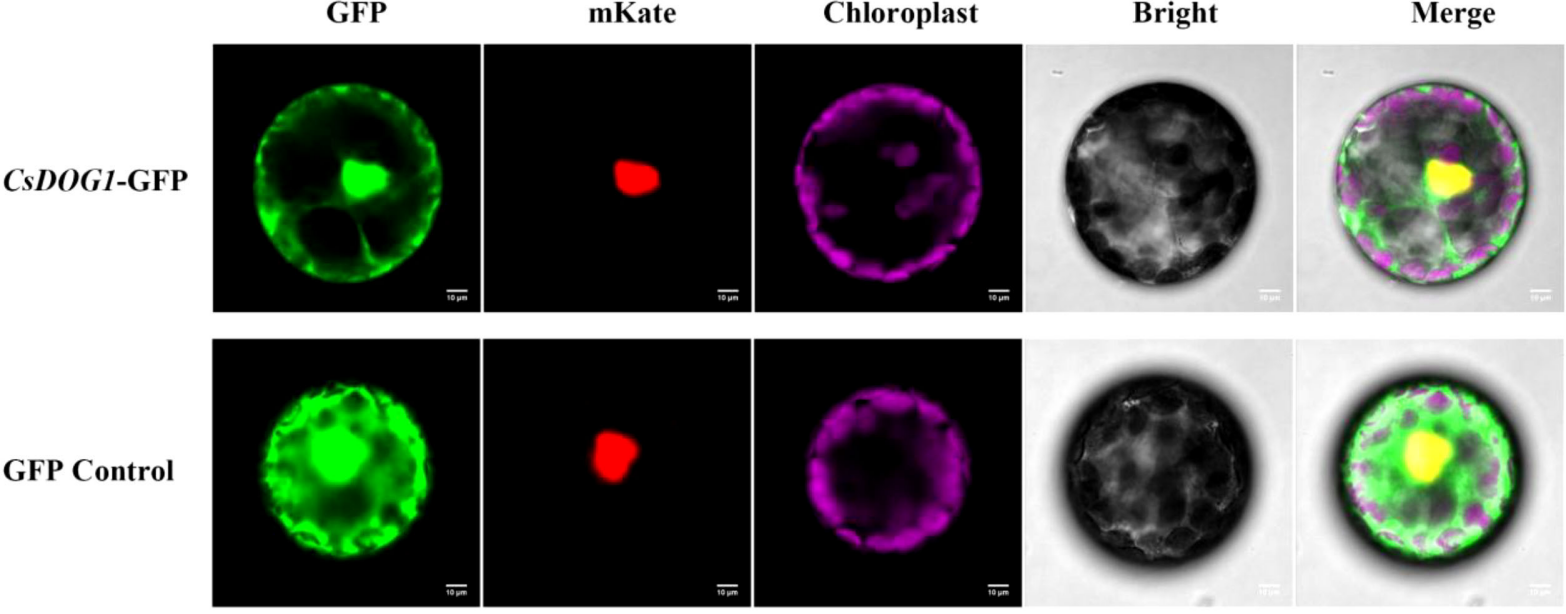
Subcellular localization of CsDOG1-GFP fusion protein via *N. benthamiana* protoplast. NLS was used as a nuclear marker. The yellow signal in the merged field represents the colocalization signal in the nucleus. Scale bars = 10 μm.

To investigate the subcellular localization of CsDOG1 protein, the fused *35S*::*CsDOG1*::*GFP* vector and the nuclear marker *35S*::*NLS*::*mKate* plasmid were co-transfected into the *N*. *benthamiana* protoplasts. Fluorescence signal assay revealed that the green fluorescence of GFP completely overlapped with the nuclear-localized red signal (**Figure 4**). Additionally, the purple autofluorescence signal specially indicated the chloroplasts. These subcellular distribution signals exhibited strong nuclear localization of the CsDOG1-GFP fusion protein, with faint but detectable fluorescence in the cytoplasm, suggesting predominant nuclear targeting with minor cytoplasmic distribution.

### Functional validation of *CsDOG1*

**Figure 5:**
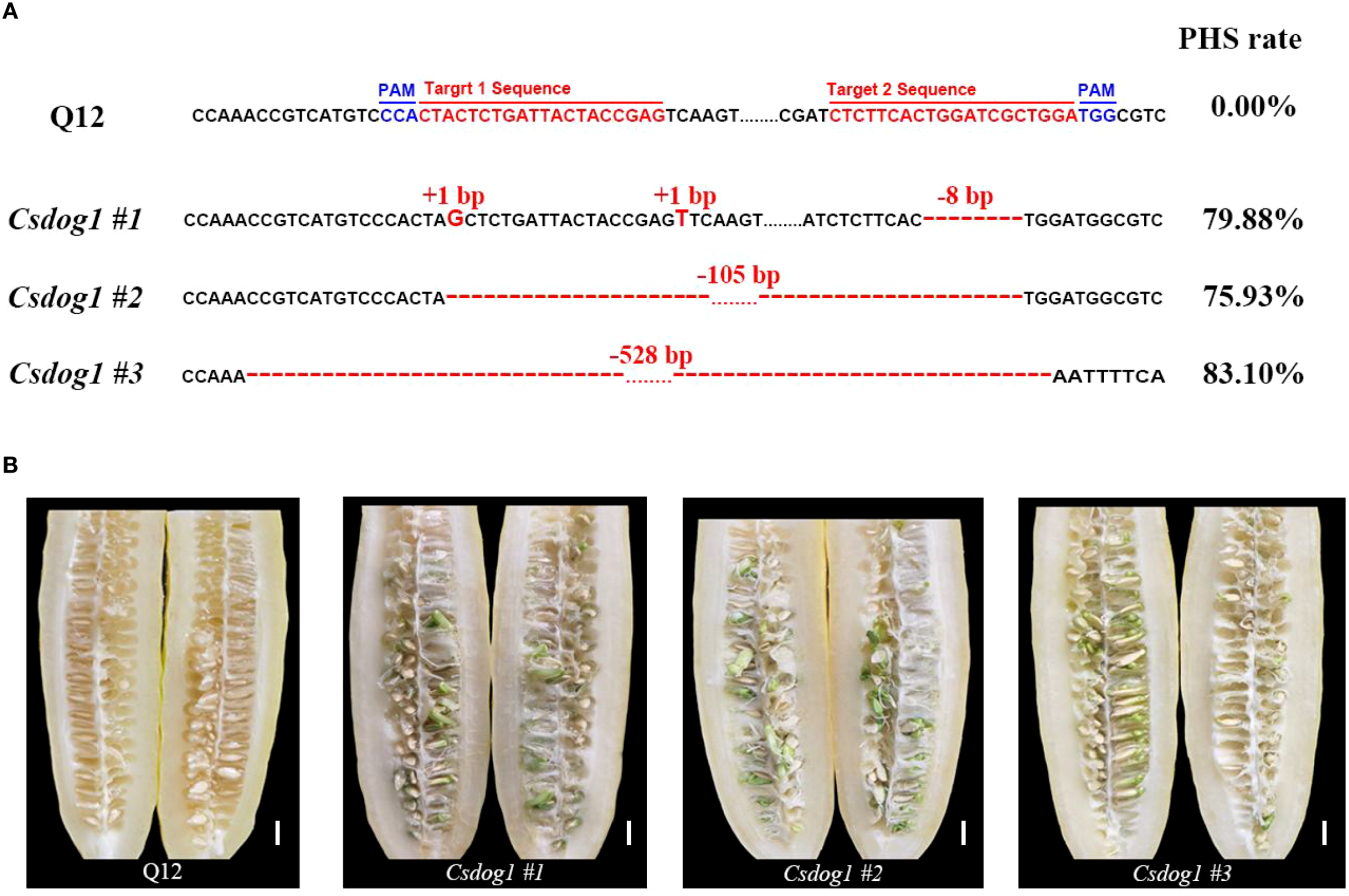
Functional validation of *CsDOG1* through CRISPR-Cas9 gene editing. **(A)** Target sequences, mutations and phenotypes for wild-type Q12 and the mutant plants, respectively. The red and blue bases individually indicate the target sites and protospacer adjacent motif (PAM) sites. Red bases and red dashes represent base insertions or deletions. Numbers indicate the specific base number of each insertion or deletion. The mean PHS rates for wild-type Q12 and mutant lines are presented in the right panel. **(B)** Phenotypic characterization of PHS in wild-type Q12 and the mutant lines. Scale bar = 1 cm.

To validate the biological function of *CsDOG1*, we employed the CRISPR/Cas9 system to perform gene editing targeting the conserved site within the first exon of *CsDOG1* gene in Q12 line. We identified three homozygous T_1_ mutant lines: *Csdog1 #1*, *Csdog1 #2* and *Csdog1 #3*. Each mutant line consisted of approximately 10 plants. Compared to the wild-type Q12, *Csdog1 #1* carried a G and a T insertion at the first target region and an 8-bp deletion in the second target region. *Csdog1 #2* and *#3* displayed a deletion of 105 bp and 528 bp, respectively (**Figure 5A**). Phenotypic characterization revealed that the PHS rate of each mutant line exceeded 75%, exhibiting severe PHS susceptibility, whereas the wild-type Q12 showed a PHS rate of 0% (**Figure 5B**). Collectively, these results confirm that *CsDOG1* is the causal gene underlying *qPHS4.1* responsible for PHS in cucumber.

## Discussion

In spermatophytes including cucumber, PHS represents a serious agronomic defect that substantially reduces seed production yield, quality and viability (Gupta et al., 2024). PHS in cereal crops predominantly occurs under prolonged rainy and high-humidity conditions during the preharvest period. Unlike cereal grains, cucumber seeds are developing and unexpectedly germinate within a specialized fruit microenvironment where the pericarp provides a physical moisture barrier (Qu et al., 2023). PHS in cucumber results from an interaction between genetic factors and environmental implications, especially for the environmental temperature, with the genetic architecture serving as the key determinant of PHS phenotypic variance. Cucumber PHS constitutes a complex trait that tightly linked to seed dormancy and is governed by multiple genes and QTLs (Qu et al., 2023). With regard to the complex trait dissection, NILs or NIL-derived populations serve as powerful genetic tools for QTL fine-mapping, as they maintain a uniform genetic background while exhibiting controlled polymorphism specifically at the target QTL interval and its flanking regions. By providing a fixed genetic background, NILs could enable the conversion of polygenic quantitative traits into Mendelian factors, effectively eliminating confounding effects from complex genetic interactions during phenotypic assessment (Gao et al., 2019, 2023). In this study, we developed a set of NILs *qPHS4.1* ^P60^ and *qPHS4.1* ^Q12^ using DNA marker-assisted selection for genetic mapping of the PHS controlling QTLs. The PHS rate of *qPHS4.1* ^P60^ line was evaluated to be 63.34%, representing only a 7.06% reduction compared to the parental P60 line (**Figure 1**). This result confirmed *qPHS4.1* as a major-effect QTL significantly associated with cucumber PHS, which was in agreement with our previous study (Cao et al., 2021). Nonetheless, *qPHS4.1* ^Q12^ line displayed a low but detectable PHS incidence (~2.03%), contrasting with the complete PHS-resistant line Q12 (0%). This result likely suggests the involvement of potential minor-effect loci contributing to PHS in cucumber.

In our initial genetic mapping study, an F_2_ population comprising 626 individuals were employed to map the *qPHS4.1* locus. The interval of *qPHS4.1* was narrowed down to a 530 kb region (Cao et al., 2021). However, this region was still too large to directly identify the candidate gene underlying *qPHS4.1*. In this study, we generated and corresponding BC_4_F_3_ backcrossing populations, and the *qPHS4.1* interval was finally narrowed down to a 69.34 kb region. Based on fine mapping and gene annotation of the QTL region, *CsaV3_4G032930* was proposed as the most likely candidate gene responsible for cucumber PHS, which was annotated to encode the DOG1 domain-containing protein. DOG1 is significantly correlated with the strength of seed dormancy in freshly harvested seeds (Nakabayashi et al., 2012). Seed dormancy and PHS represent a pair of antagonistic traits in seed physiology, exhibiting an inverse correlation between dormancy intensity and PHS susceptibility (Zhang et al., 2014; Xu et al., 2019). Varieties with high levels of dormancy typically demonstrate high PHS resistance, whereas those with weak dormancy are susceptible to PHS.

As a key dormancy regulator, *DOG1* regulates seed dormancy by systematically controlling the expressions of hormone metabolism genes, and coordinating hormonal signaling networks especially for ABA and GAs (Li et al., 2022). In *Arabidopsis*, *DOG1* quantitatively regulates seed dormancy by modulating the ABA/GA ratio in dormant seeds through transcriptional networks (Footitt et al., 2020). The *OsDOG1L-3* gene participates in the ABA pathway by upregulating the expression of ABA-related genes and increasing ABA content to establish seed dormancy in rice (Wang et al., 2020). In PHS-sensitive P60, a mutation of 3-bp deletion was detected in Exon 2 of *CsDOG1* (**Figure 2D**; **Supplementary Figure S2**), which resulted in a loss of a conserved asparagine residue (-N) in the encoded protein sequence. We conducted comprehensive DNA sequence analysis of *CsDOG1* gene across 40 diverse cucumber accessions and evaluated their PHS phenotypes. The 3-bp InDel within the *CsDOG1* gene was found to be consistently associated with the PHS phenotype. The phenotypic characteristics and genotypic variations showed strong concordance with that in the BC_4_F_2_ population (**Table 1**). This combined evidence strongly suggests that *CsDOG1* is the causal gene underlying the *qPHS4.1* locus. To functionally validate the role of *CsDOG1*, we generated mutant lines by targeting the conserved Exon1, which is shared by the gene’s two representative transcripts (Guan et al., 2024). All the three independent mutant lines exhibited severe PHS susceptibility. These data strongly demonstrated that *CsDOG1* is the causal gene that enables seed dormancy and confers PHS-resistance to cucumber seeds, as loss-of-function mutations significantly result in severe PHS.

Expression profiling revealed that *CsDOG1* is predominantly expressed in developing and mature ungerminated seeds, whereas seed germination could significantly decrease the expression. Comparative sequence analysis revealed that the *CsDOG1* promoter sequence was conserved between P60, Q12, and 40 natural cucumber germplasms, suggesting that differential *CsDOG1* gene expression is not caused by variation in the promoter region. In wheat, *TaDOG1* genes exhibited distinct expression patterns in different tissues, primarily expressing in the stem tissue (Ko et al., 2024). Nonetheless, high expression levels of *TaDOG1-1-1A*, *-1B*, and *-1D* were observed during the grain-filling stage, especially during hard dough and ripening stages (Ko et al., 2024). In the present study, much lower or nearly no expression was detected in the other tissues of cucumber, indicating that the cucumber *CsDOG1* is a strict seed-specifically expressed gene, in consistence with the expression pattern of *AtDOG1* (Bentsink et al., 2006). Bioinformatic analysis identified several motifs in the promoter that associated with tissue-specific expression, ABRE elements for ABA responsiveness and transcriptional promoter or enhancer. The tissue-specific expression motifs found in promoter further corroborated the seed-specific expression pattern of *CsDOG1*. The coexistence of seed-specific motifs and ABA-responsive elements in the *CsDOG1* promoter suggests integrated regulation by both developmental and environmental cues. In addition, comparative expression analysis revealed significantly higher *CsDOG1* transcript levels in P60 across multiple tissues relative to Q12. Considering that P60 is very sensitive to PHS, we speculate that the amino acid deletion in Csdog1 may generate a loss-of-function allele, impairing dormancy establishment and triggering PHS.

In conclusion, we identified the *CsaV3_4G032930* (*CsDOG1*) gene underlying *qPHS4.1* locus controlling PHS in cucumber through backcrossing populations. Expression profiling revealed that *CsDOG1* is a seed-specific gene predominantly localized in the nucleus. Functional knockout of *CsDOG1* led to extreme PHS susceptibility, confirming its critical role in PHS control. Our findings elucidate the theoretical basis of cucumber PHS variation and provide valuable genetic resources for PHS-resistant breeding programs.

## Data Availability

The raw datasets are stored in NCBI. The Q12 and P60 resequencing raw datasets are SRR13637896 and SRR13637895, respectively. The analyzed data during this study are included in this published article and its **Supplementary information files**.

## Supplementary material

Supplementary Table 1 Primers of the sites used in *qPHS4.1* interval.

Supplementary Table 2 Genotypes and PHS phenotypes of the 187 recombinant individuals in BC_4_F_2_ population.

Supplementary Table 3 Genotypes and phenotypes of 40 natural cucumber accessions at the target 3-bp InDel site in *CsDOG1* gene.

Supplementary Table 4 Primers for gene cloning and qRT-PCR.

Supplementary Table 5 Putative cis-acting regulatory elements and motifs in *CsDOG1* promoter.

Supplementary Table 6 Genotypes and PHS phenotypes of the homozygous recombinants in BC_4_F_3_ population.

Supplementary Figure 1 Several codominant InDel markers developed within the *qPHS4.1* interval.

Supplementary Figure 2 Alignments of partial coding sequences **(A)** and their derived amino acid sequences **(B)** between *CsDOG1* and *Csdog1*.

Supplementary Figure 3 Promoter sequence alignment of *CsDOG1* gene in P60 and Q12.
