# Supplementary material for: Fine mapping of the major-effect QTL qPHS4.1 revealed a CsDOG1 gene controlling pre-harvest sprouting in cucumber (Cucumis sativus L.)
Source: Front Plant Sci. 2025 Oct 9;16:1701268. doi: 10.3389/fpls.2025.1701268 (PMC12547290; doi:10.3389/fpls.2025.1701268)
Supplement: Supplementary Figure 1 — Several codominant InDel markers developed within the qPHS4.1 interval. [file Table1.docx]

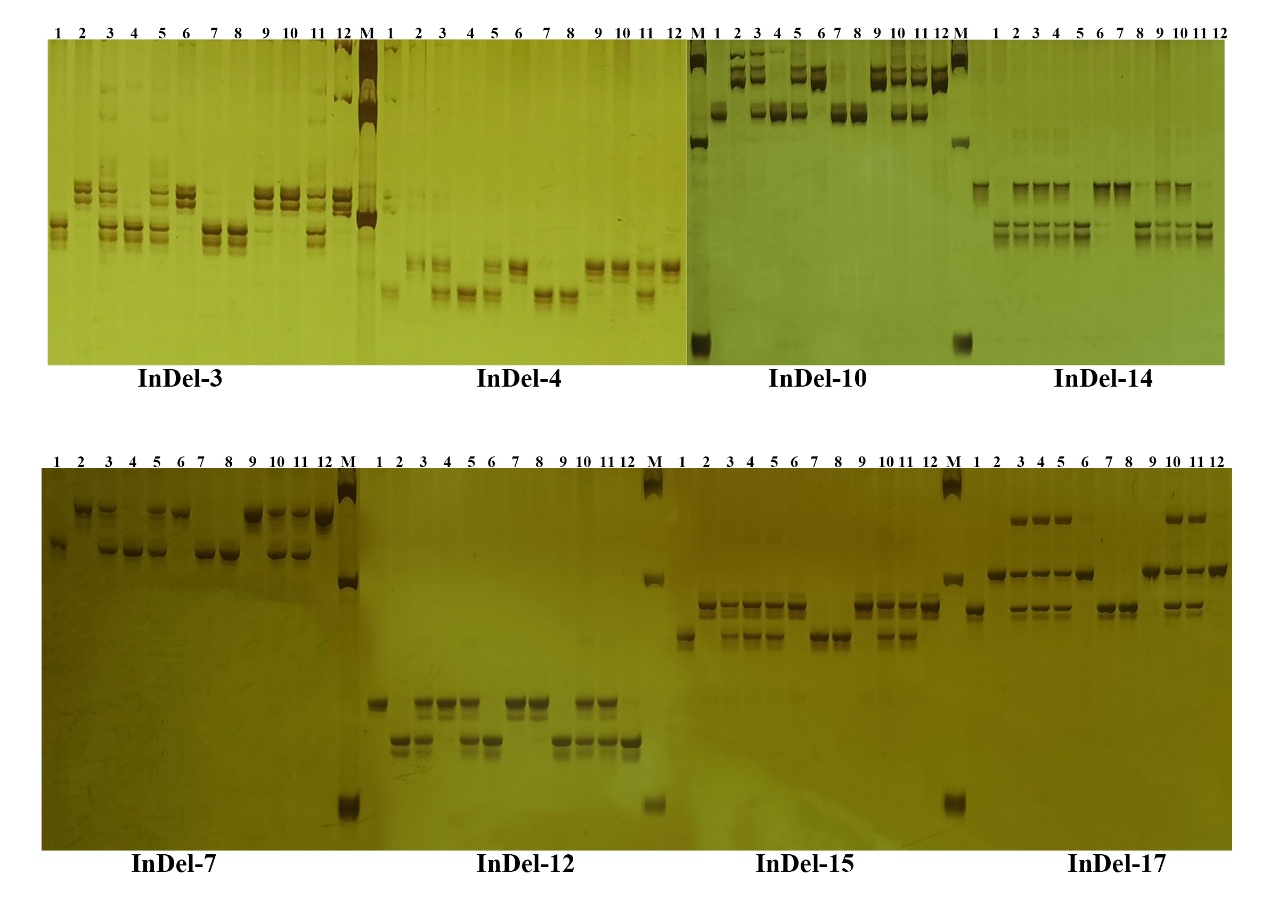


Fig. S1 Several codominant InDel markers developed within the *qPHS4.1* interval. M: marker; 1: parent P60; 2: parent Q12; 3: F_1_ individual; 4-12: BC_4_F_2_ individuals.


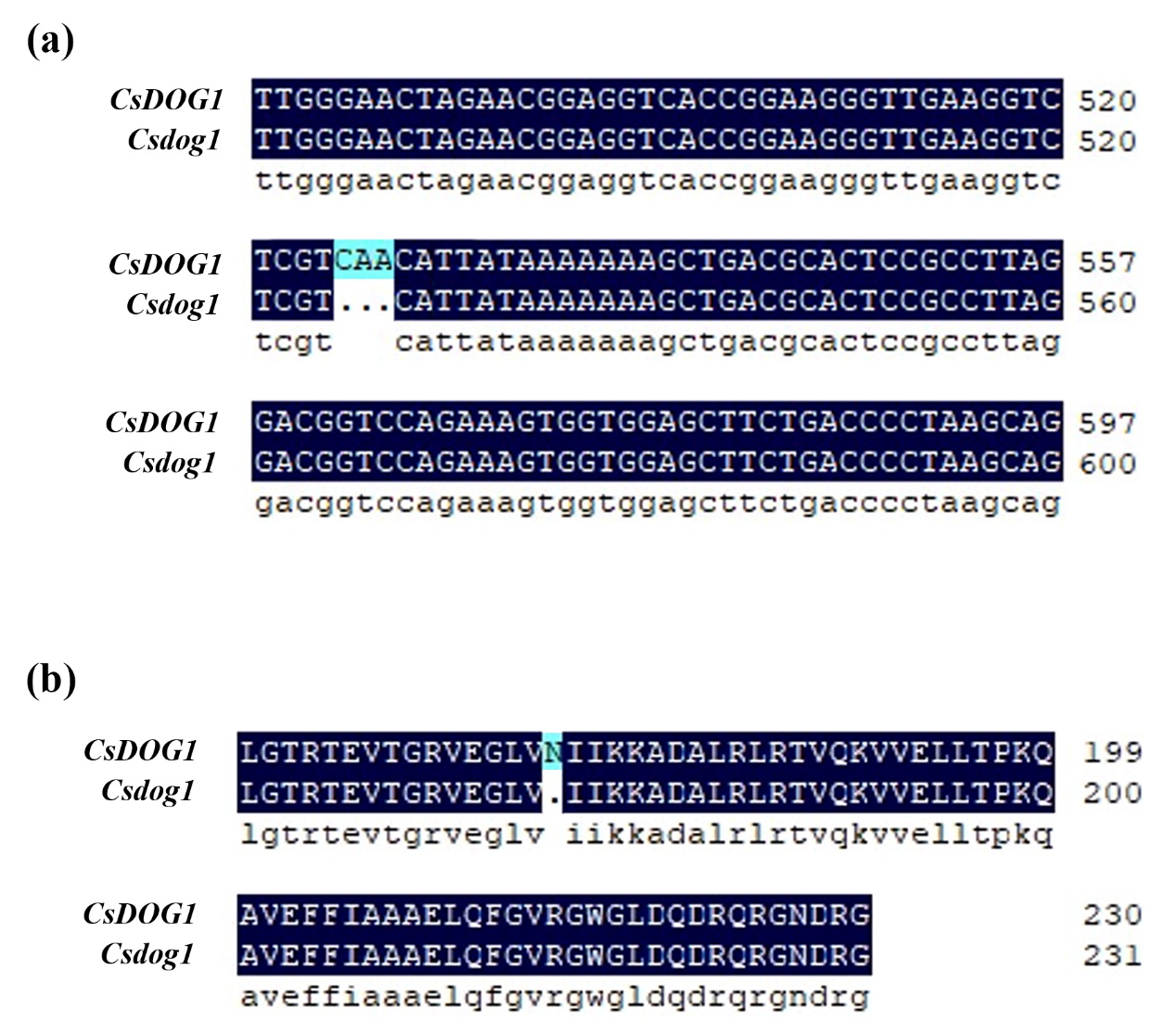


Fig. S2 Coding sequence (a) and the derived amino acid sequence (b) alignments between *CsDOG1* and *Csdog1*.


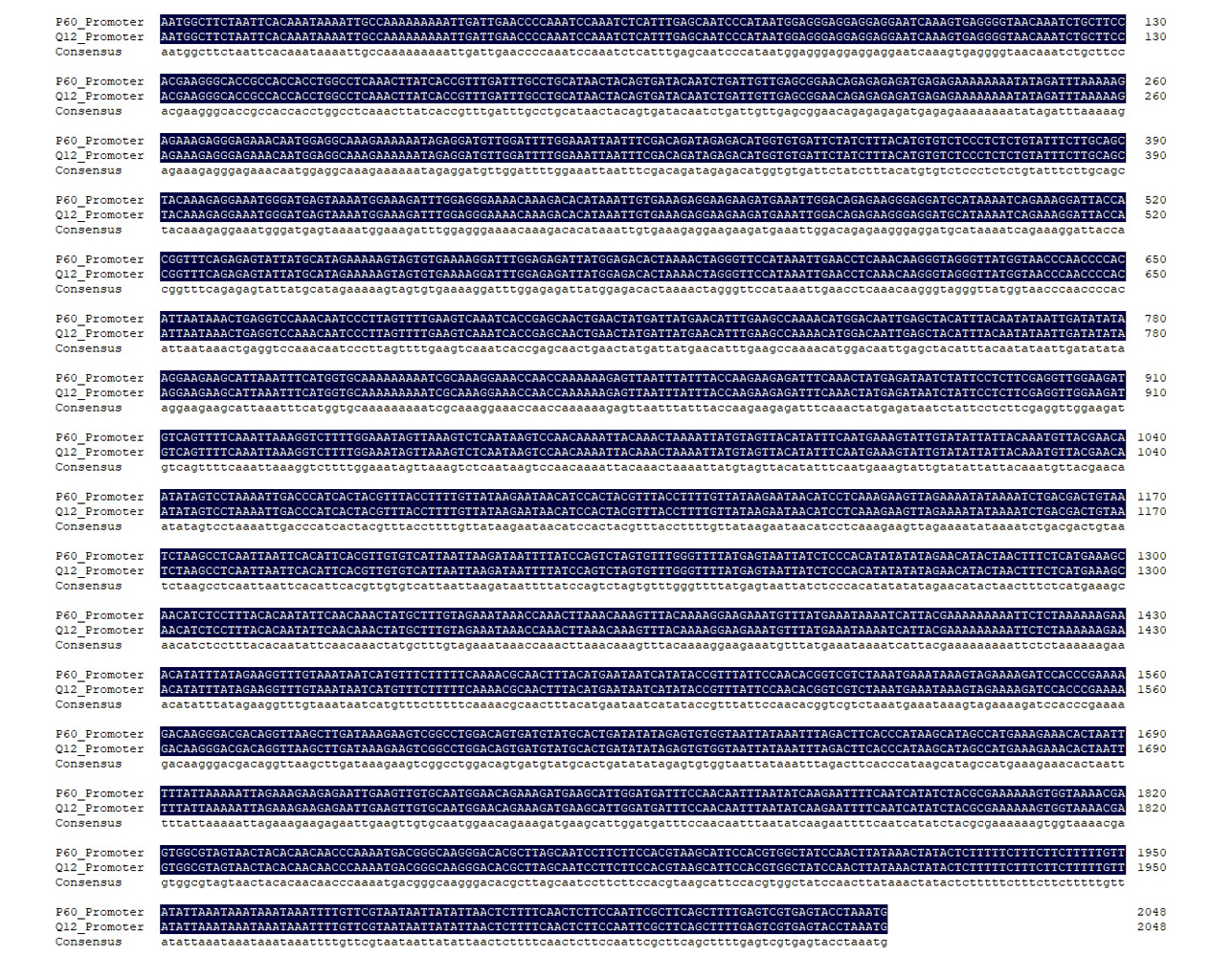


Fig. S3 Promoter sequence alignment of *CsDOG1* gene in P60 and Q12.
